# The role of microRNAs in depression

**DOI:** 10.3389/fphar.2023.1129186

**Published:** 2023-03-29

**Authors:** Ruidong Ding, Dingyuan Su, Qian Zhao, Yu Wang, Jia-Yi Wang, Shuangyu Lv, Xinying Ji

**Affiliations:** ^1^ Institute of Molecular Medicine, Henan International Joint Laboratory for Nuclear Protein Regulation, School of Basic Medical Sciences, Henan University, Kaifeng, Henan, China; ^2^ San-Quan College, Xinxiang Medical University, Xinxiang, Henan, China; ^3^ Kaifeng Key Laboratory for Infectious Diseases and Biosafety, Kaifeng, Henan, China; ^4^ Faculty of Basic Medical Subjects, Shu-Qing Medical College of Zhengzhou, Zhengzhou, Henan, China

**Keywords:** microRNA, depression, brain, biomarker, MDD

## Abstract

Major depressive disorder (MDD) is a psychiatric disorder with increasing prevalence worldwide. It is a leading cause of disability and suicide, severely affecting physical and mental health. However, the study of depression remains at an exploratory stage in terms of diagnostics and treatment due to the complexity of its pathogenesis. MicroRNAs are endogenous short-stranded non-coding RNAs capable of binding to the 3’untranslated region of mRNAs. Because of their ability to repress translation process of genes and are found at high levels in brain tissues, investigation of their role in depression has gradually increased recently. This article summarizes recent research progress on the relationship between microRNAs and depression. The microRNAs play a regulatory role in the pathophysiology of depression, involving dysregulation of monoamines, abnormalities in neuroplasticity and neurogenesis, hyperactivity of the HPA axis, and dysregulation of inflammatory responses. These microRNAs might provide new clue for the diagnosis and treatment of MDD, and the development of antidepressant drugs.

## 1 Introduction

### 1.1 MicroRNAs

MicroRNAs (miRNAs) are short-stranded endogenous non-coding RNA molecules with a length of 19–25 nucleotides. A single microRNA can target hundreds of mRNAs and influence the expression of many genes (Friedman et al., 2009; Lu and Rothenberg, 2018). It is now established that about 70% of the known microRNAs are expressed in the brain and play critical roles in brain development through key signaling pathways involving synapse formation, neuronal plasticity, nerve growth, *etc*. MicroRNAs are endogenously encoded in the mammalian genome and are transcribed in the nucleus as primary transcripts (pri-miRNAs) which are hundreds of nucleotides in length. Pri-miRNAs are then trimmed into precursor microRNAs (pre-miRNAs) within the nucleus by DiGeorge syndrome critical region 8 (DGCR8) and Drosha. After processing in the nucleus, pre-miRNA transcripts are transported to the cytoplasm *via* the transporter Exportin-5 (XPO5). Pre-miRNAs are further processed in the cytoplasm by the enzyme Dicer into approximately 22 nucleotide-long RNA duplexes. The RNA duplexes are incorporated into the RNA-induced gene silencing complex (RISC), and further processed to form mature microRNAs (Zurawek and Turecki, 2021). RISC binds to the 3’untranslated region (3′UTR) of target mRNAs to induce targeted mRNA degradation or translational repression, thereby controlling gene expression at the post-transcriptional level.

### 1.2 Molecular pathophysiology of depression

Major depressive disorder (MDD) is a common illness that severely limits psychosocial functioning and diminishes quality of life (Malhi and Mann, 2018). MDD causes emotional changes in patients, as well as depressed mood and anhedonia, and it can lead to several psychiatric symptoms, including cognitive impairment (Hu et al., 2017). Although, there has been considerable research looking at the pathophysiology of major depressive disorder (MDD), no single mechanism can satisfactorily and completely explain all aspects of the disorder. There are several hypotheses regarding the molecular mechanisms involved in depression, including the monoamine hypothesis, hypothalamic-pituitary-adrenal (HPA) axis, neuroplasticity and neurogenesis, epigenetics, and inflammation. The monoamine hypothesis reveals that the pathophysiological basis leading to depression is due mostly to a decrease in monoamine neurotransmitters (e.g., serotonin). Evidence from clinical trials of some tricyclic antidepressants and monoamine oxidase inhibitors (MAOIs) have provided the basis for this hypothesis (Segal et al., 1974; Delgado et al., 1990; Willner et al., 2013). Hyperactivity of the HPA axis can lead to the stimulation of glucocorticoids and cortisol secretion, which may contribute to the development of depression (Goodyer et al., 2000; Harris et al., 2000). Notably, alterations of the HPA axis have also been associated with impairment of cognitive function (Keller et al., 2017). Stress-mediated inflammation and HPA axis dysfunction can lead to an alteration in neuroplasticity at the cellular level (Egeland et al., 2015). The neurogenesis process is controlled by regulatory proteins, such as brain-derived neurotrophic factor (BDNF), and peripheral BDNF has been found to be downregulated in patients with MDD (Molendijk et al., 2014). Epigenetics, the interaction of genes and the environment, plays a role in the alteration of brain neurobiology, and the effect of epigenetics can set the stage for the development of MDD (Penner-Goeke and Binder, 2019). In addition, peripheral cytokines can directly act on neurons and support cells and subsequently contribute to the development of depression (Miller and Raison, 2016). This hypothesis is supported by a role for some non-steroidal anti-inflammatory drugs in the treatment of depression (Leonard, 2018). Patients with autoimmune diseases and severe infections both have persistent activation of the immune system, causing high levels of cytokine production in the periphery. Such changes will cause changes in the patient’s central nervous system function, which in turn will lead to the occurrence and development of depression. This mechanism may explain why individuals with autoimmune diseases and severe infections are more likely to become depressed.

Up to now, first-line antidepressant drugs and other selected drugs in the clinic have low effectiveness, variable tolerance, adverse effects, and other disadvantages. Furthermore, large variations in therapeutic effects exist among individual drugs (Malhi and Mann, 2018). Our current understanding of microRNAs is continuing to increase partly, because of their high expression levels in the brain and their role in the regulation of neuronal plasticity and other functions. Recently, researchers focused on a role for microRNAs in the etiology of MDD. In this review, we have summarized the roles and mechanisms of microRNAs-mediated gene expression in the pathophysiological process of MDD. The role of each microRNA implicated in depression will be described as it relates to the different hypotheses of depression. In addition, this review could provide an attractive clue and potential targets to help diagnose and treat depression, as well as to assist in antidepressant drug development.

## 2 Expression and regulation of microRNAs in clinical samples of depression

Many studies have confirmed that the level of microRNAs expression is associated with the onset of depression. These studies include both human and animal experiments. Postmortem human experiments were carried out to examine the expression levels of microRNAs in the prefrontal cortex, amygdala and other regions, as well as the levels and identity of their downstream target genes and protein products (As shown in Table 1). These human studies also looked at peripheral whole blood, serum, exosomes, and other tissues. The animal experiments were performed to detect microRNAs, and their downstream target genes and protein expression in the hippocampus and other tissues in rodents with depression-like symptoms (Table 2). The depression-like symptoms were induced by chronic unpredictable mild stress (CUMS) and this successful animal model was confirmed using behavioral tests, including sucrose preference test, forced swim test, and elevated plus maze test. According to the literature, microRNAs such as miR-124-3p, miR-128-3p, miR-139-5p, and miR-144-5p have been shown to play a significant role in different pathophysiological mechanisms of depression, which will be described in the corresponding sections of the text according to their different roles.

**TABLE 1:** Summary of researches on the changes in the levels of microRNAs and their target genes in MDD patients.

References	Sample sources	microRNA	Regulation MDD vs. HC	Targeted gene	Expression of target gene
Gorinski et al. (2019)	Brodmann Area 9(BA9)	miR-30a, miR-30e	Up	ZDHHC21	Down
		miR-200a	Down		
Wingo et al. (2020)	Brodmann Area 9(BA9)/Brodmann Area 46(BA46)	miR-484, miR-26b-5p, miR-30d-5p, miR-197-3	Down		
Smalheiser et al. (2012)	Brodmann Area 9(BA9)	miR-20b, miR-20a, miR-34a, miR-34b	Down	VEGFA	
		miR-34a	Down	Bcl-2	Down
		miR-148b	Down	DNMT3B	Up
Maussion et al. (2012)	Brodmann Area 10(BA10)	miR-185	Up	TrkB-T1	Down
Smalheiser et al. (2014)	Dorsolateral Prefrontal Cortex (BA10)	miR-508-3p, miR-152	Down		
Wang et al. (2018a)	Dorsolateral Prefrontal Cortex (BA10)	miR-19a-3p	Up	Tumor Necrosis Factor-α(TNF-α)	Up
		miR-20a-5p, miR-92a-1-3p	Down		
	Peripheral Blood Mononuclear Cells (PBMC)	miR-19a-3p	Up	Tumor Necrosis Factor-α(TNF-α)	Up
Fiori et al. (2021)	Brodmann Area 24(BA24)	miR-323a-3p (miR-204-5p, miR-331-3p)	Up	ERBB4	Down
	Cerebral lateral habenula	miR-323a-3p (miR-320b-3p, miR-331-3p)	Up	ERBB4	Down
Wang et al. (2018b)	Brodmann Area 44(BA44)	miR-124-3p	Down	DDIT4	Up
				SP1	Up
Torres-Berrio et al. (2017)	Brodmann Area 44(BA44)	miR-218	Down	DCC	Up
Lopez et al. (2014a)	Brodmann Area 44(BA44)	miR-320c, miR-34c-5p	Up	SAT1	Down
		miR-320c, miR-139-5p	Up	SMOX	Down
		miR-195	Up		
Roy et al. (2017a)	Brodmann Area 46(BA46)	miR-124-3p	Up	GRIA3, GRIA4, NR3C1	Down
	Serum	miR-124-3p	Up	GRIA3, GRIA4, NR3C1	Down
Lopez et al. (2017)	Ventrolateral Prefrontal Cortex (BA47)	miR-146a-5p, miR-146b-5p, miR-425-3p, miR-24-3p	Up		
Lopez et al. (2014b)	Ventrolateral Prefrontal Cortex (BA47)	miR-1202	Down	GRM4	Up
Yoshino et al. (2020)	Anterior Cingulate Cortex (ACC)	117 microRNAs (4.16%)	Up		
		54 microRNAs (2.13%)	Down		
Azevedo et al. (2016)	Anterior Cingulate Cortex (ACC)	miR-34a	Down	NCOA1	Up
		miR-184	Down	NCOR2	Down
		miR-34a, miR-184	Down	PDE4B	
Maheu et al. (2015)	Basolateral Amygdala	miR-511	Up	GFRA1	Down
Roy et al. (2020)	Cerebral Amygdala	miR-128-3p	Up	DVL1, LEF1, WNT5b	Down
Roy et al. (2017b)	Locus Coeruleus	miR-17-5p, miR-20b-5p, miR-106a-5p, miR-330-3p, miR-541-3p, miR-582-5p, miR-890, miR-99-3p, miR-550-5p, miR-1179	Up	GRIK1	Up
		miR-409-5p, let-7g-3p, miR-1197	Down	RELN, GSK-3β, MAOA, CHRM1, PLCB1	Down
Aschrafi et al. (2016)	Midbrain	miR-326	Down	Urocortin 1 (Ucn1)	Up
Issler et al. (2014)	Raphe Nuclei (RN)/Whole Blood	miR-135a	Down	Htr1a, Slc6A4	Up
Morgunova and Flores (2021)	Prefrontal Cortex (PFC)	miR-218-5p	Down	DCC	Up
Liu et al. (2021c)	Peripheral Blood Mononuclear Cells (PBMC)	miR-374b, miR-10a	Down		
Hung et al. (2019)	Peripheral Blood Mononuclear Cells (PBMC)	let-7e, miR-21-5p, miR-146a, miR-155	Down	IL-6	Up
	Monocytes	miR-146a, miR-155	Down		
Sun et al. (2016)	Peripheral Blood Mononuclear Cells (PBMC)	miR-34b-5p, miR-34c-5p	Up	NOTCH1	Down
He et al. (2016)	Peripheral Blood Mononuclear Cells (PBMC)	miR-124	Up		
Vaisvaser et al. (2016)	Peripheral Blood Mononuclear Cells (PBMC)	miR-29c	Up		
Gecys et al. (2022)	Plasma	let-7e-5p, miR-125a-5p	Up		
Roumans et al. (2021)	Plasma	let-7b-5p	Down	ERK1/2	Down
Sundquist et al. (2021)	Plasma	miR-144-5p	Down	21 Inflammatory Proteins	Up
				15 Inflammatory Proteins	Down
Chen et al. (2020)	Plasma	miR-19b-3p	Down		
Zhang et al. (2020a)	Plasma	miR-134	Down		
Mendes-Silva et al. (2019)	Plasma	miR-184	Down		
Van der Auwera et al. (2019)	Plasma	let-7g-5p, miR-103a-3p, miR-107, miR-142-3p	Down		
Fang et al. (2018)	Plasma	miR-132, miR-124	Up		
Camkurt et al. (2015)	Plasma	miR-451a	Up	SLC17A7	Down
		miR-320a	Down	GRIN2A, DISC1	Up
		miR-17-5p, miR-223-3p	Up		
Al-Rawaf et al. (2021)	Serum	miR-34a-5p, miR-124	Up	iNOS, Cortisol	Up
		miR-135, miR-451-a	Down	SOD2, CAT,5-HT	Down
Liu et al. (2021d)	Serum/Cerebrospinal Fluid	miR-199a-5p	Up	WNT2	Down
	Hippocampus	miR-199a-5p	Up	WNT2	Down
Feng et al. (2019)	Serum	miR-221-3p	Up	IRF2	Down
Gheysarzadeh et al. (2018)	Serum	miR-16, miR-135a, miR-1202	Down		
Kuang et al. (2018)	Serum	miR-451a	Down		
		miR-34a-5p, miR-221-3p	Up		
He et al. (2021)	Peripheral Blood	miR-9	Up		
Sun et al. (2020)	Peripheral Blood	miR-34c-5p	Up		
Zhao et al. (2019)	Peripheral Blood	pmiR-chr11	Up	BRPF1	Down
Qi et al. (2018)	Peripheral Blood	miR-132	Up		
Wang et al. (2018c)	Peripheral Blood	miR-155	Up	SIRT1	Down
Liu et al. (2016)	Peripheral Blood	miR-132	Up		
Li et al. (2021a)	Plasma Exosome	miR-335-5p	Up		
		miR-1292-3p	Down		
Liang et al. (2020)	Serum Exosome	miR-139-5p	Up		
Xian et al. (2022)	Serum Exosome	miR-9-5p	Up		
Wei et al. (2020)	Blood Exosome	miR-139-5p	Up		
Mizohata et al. (2021)	Neural Extracellular Vesicles (NEVs) in Blood	miR-17-5p	Up		

**TABLE 2:** Summary of researches on the changes in the levels of microRNAs and their target genes in experimental animals induced to develop depression.

References	Sample sources	microRNA	Regulation MDD vs. HC	Targeted gene	Expression of target gene
Kavuran Buran et al. (2022)	Hippocampus	miR-135a-5p, miR-135b-5p, miR-6334, miR-203a-3p, miR-296-5p, miR-6320	Up		
	Prefrontal Cortex (PFC)	miR-135a-5p, miR-135b-5p	Up		
		miR-484, miR-501-3p, miR-296-5p, miR-361-3p	Down		
Kim et al. (2022)	Prefrontal Cortex (PFC)	miR-329, miR-362	Up	Baiap3	Down
Yoshino et al. (2022)	Prefrontal Cortex (PFC)	miR-218a-5p	Up	DTWD1, BNIP1, METTL22, SNAPC1, HDAC6	Down
Huang et al. (2021a)	Prefrontal Cortex (PFC)/Hippocampus	miR-23a-5p	Up		
		miR-98-5p, miR-3968	Down		
Gorinski et al. (2019)	Brodmann Area 9(BA9)	miR-30a, miR-30e	Up	ZDHHC21	Down
		miR-200a	Down		
Torres-Berrio et al. (2017)	Brodmann Area 44(BA44)	miR-218	Down	DCC	Up
Roy et al. (2017a)	Brodmann Area 46(BA46)	miR-124-3p	Up	GRIA3, GRIA4, NR3C1	Down
	Serum	miR-124-3p	Up	GRIA3, GRIA4, NR3C1	Down
Lopez et al. (2017)	Ventrolateral Prefrontal Cortex (BA47)	miR-146a-5p, miR-146b-5p, miR-425-3p, miR-24-3p	Up		
Liu et al. (2021a)	Hippocampus	miR-883b-3p	Down	Adcy1, Nr4a2	Up
		miR-377-3p	Down	Six4, Stx16, Ube3a	Up
Si et al. (2021)	Peripheral Samples/Hippocampus	miR-212	Up	Nuclear Factor I-A (NFIA)	Down
Huang et al. (2021b)	Hippocampus	miR-139-5p	Down	Phosphodiesterase 4D (PDE4D)	Up
				p-CREB, BDNF	Down
Lan et al. (2021)	Hippocampus	miR-204-5p	Down	RGS12	Up
Liu et al. (2021b)	Hippocampus	miR-383	Up	WNT2	Down
Liu et al. (2021d)	Serum/Cerebrospinal Fluid	miR-199a-5p	Up	WNT2	Down
	Hippocampus	miR-199a-5p	Up	WNT2	Down
Li et al. (2021c)	Hippocampus Dentate Gyrus	miR-26a-3p	Up	PTEN	Down
Li et al. (2021b), Shen et al. (2021)	Hippocampus CA1 Region/Hippocampus Dentate Gyrus	miR-211-5p	Down	Dyrk1A	Up
Qin and Li (2022)	Hippocampus	miR-124-3p	Up	STAT3, Bcl-2	Down
				Bax	Up
Su et al. (2022)	Hippocampus	miR-139-5p	Up	NR3C1	Down
Li et al. (2022)	Hippocampus	miR-497a-5p	Up	NR3C1	Down
Mingardi et al. (2021)	Hippocampus	miR-9-5p	Down	REST	Up
Ding et al. (2021)	Peripheral Blood	miR-135a	Down		
	Peripheral Blood/Hippocampus	miR-135a	Down	TLR4	Up
				IL-1β, IL-6, TNF-α	Up
				Bax Protein	Up
				Bcl-2 Protein	Down
Roy et al. (2020)	Cerebral Amygdala	miR-128-3p	Up	DVL1, LEF1, WNT5b, Snail1, Arpp21	Down
Volk et al. (2016)	Cerebral Amygdala	miR-15a	Up	FKBP51	Down
Aschrafi et al. (2016)	Midbrain	miR-326	Down	Urocortin 1 (Ucn1)	Up
Issler et al. (2014)	Raphe Nuclei (RN)/Whole Blood	miR-135a	Down	Htr1a, Slc6A4	Up
Fei et al. (2020), Huang et al. (2022)	Brain Microglia	miR-29b-3p	Down	MMP2	Up
Wang et al. (2021)	Neural Stem Cells (NSC)	miR-34a-5p	Up	Tia1	Down

## 3 Involvement of microRNAs in the pathophysiology of depression

### 3.1 MicroRNAs are involved in the pathophysiology of depression induced by the dysregulation of monoamines

Monoamine neurotransmitter (serotonin, noradrenaline, and dopamine) dysregulation is considered the most likely cause of MDD, and most of the drugs used in the clinic for the treatment of MDD are based on this principle. Monoamine-based antidepressants were the first drugs developed for the treatment of MDD (Elias et al., 2022). The monoamine hypothesis of depression has been applied for nearly six decades ago (Coppen et al., 1965) and the classical doctrine holds that monoamines are depleted and chronically below normal levels in the brains of patients with MDD (Shaw et al., 1967). This hypothesis is corroborated by the pharmacological mechanism of action of monoamine oxidase (MAO) inhibitors, tricyclic antidepressants, and selective serotonin reuptake inhibitors in MDD patients (Hillhouse and Porter, 2015). In 1996, Heninger et al. (1996) revised the monoamine doctrine to suggest that monoamine depletion may play more of a role, thereby affecting nervous system functions, or it must be present in the environment of a stressor to cause MDD. They provided a theoretical basis for investigating the role of microRNAs in MDD.

**FIGURE 1:**
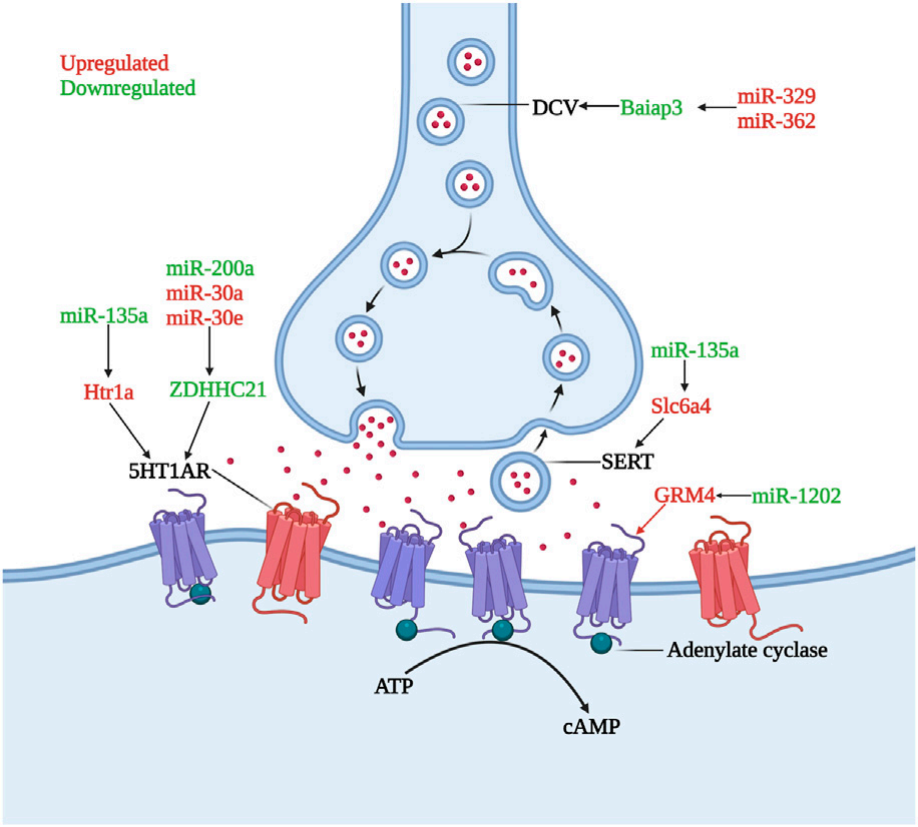
Role of microRNAs in serotonin dysregulation. The inhibitory receptor 5HT1AR is hyperactivity or increased under the influence of miR-135a, miR-200a, miR-30a, and miR-30e, lead to the occurrence and development of depression. DCV and SERT undergo quantitative abnormalities under the influence of miR-329, miR-362 and miR-135a, causing dysregulation of monoamine transmitter secretion and reuptake. GRM4, whose transcription is increased by miR-1202 downregulation, can regulate monoamine neurotransmitter transmission (Created with BioRender.com).

Gorinski et al. (2019) found that a decrease in miR-200a expression or an increase in miR-30a and miR-30e expression led to a decrease of ZDHHC21 expression in humans and animal models. ZDHHC21, a palmitoyl acyltransferase, was identified as the major enzyme involved in the palmitoylation of the 5HT1AR and the decrease in the palmitoylation of 5HT1AR resulted in inhibition of adenylate cyclase and subsequent decrease of cAMP levels resulting in the occurrence of MDD. The downregulated miR-135a was shown to promote the translation of the *Htr1a* and *Slc6a4* genes in MDD patients (Issler et al., 2014) and the upregulation of the inhibitory 5HT1a receptor (5HT1AR), encoded by the *Htr1a* gene, and 5HT transporter (SERT), encoded by the *Slc6a4* gene, contributed to aberrant monoamine neurotransmitters in patients with depression (Issler et al., 2014). DCC (Deleted in Colorectal Cancer) drives prefrontal cortex maturity by determining DA targets early in life, for example, in rats, signaling within dopamine neurons in the juvenile VTA determines the extent of innervation of the PFC (Torres-Berrio et al., 2017). Whereas miR-218 was shown to be upregulated in BA44 in MDD patients and led to a significant decrease in DCC expression levels. In rats, who had experienced chronic social defeat stress paradigms also showed the same changes (Torres-Berrio et al., 2017). MiR-1202 was found to be differentially expressed in MDD patient ventrolateral prefrontal cortices, with upregulated GRM4 expression (Lopez et al., 2014b). GRM4 is expressed throughout the brain, with predominant expression sites at presynaptic and postsynaptic membranes, where it regulates glutamatergic, dopaminergic, GABAergic, and serotonergic neurotransmission (Lopez et al., 2014b). The increased expression of miR-329 and miR-362 in the PFC of MDD patients caused downregulation of Baiap3 (brain specific angiogenesis inhibitor 1-associated protein 3), which subsequently induced defective dense core vesicles (DCVs) transport and reduced serotonin exocytosis (Kim et al., 2022). In both the central nervous system and endocrine systems, DCVs are essential for peptidergic and aminergic signaling (Persoon et al., 2018) (Figure 1).

### 3.2 MicroRNAs are involved in the pathophysiological processes of depression related to neuroplasticity and neurogenesis abnormalities

Neuroplasticity is a fundamental process by which the brain acquires information and produces appropriately adaptive responses in relevant environments. Thus, dysfunction in neuroplasticity and neurogenesis may contribute to the pathophysiology of MDD (Duman, 2002). Multiple signaling pathways are involved in this process. For example, Wnt signaling pathway plays a role in neurogenesis, synapse formation, synaptic transmission, and dendritic arborization in the hippocampus (Wayman et al., 2006; Gogolla et al., 2009). The mTOR signaling pathway is involved in the pathophysiology of MDD through the P70S6K/eIF4B pathway (Jernigan et al., 2011). Abnormalities in BDNF, glutamate receptors, VEGF signaling, and long-term potentiation (LTP) pathways also contribute to the pathophysiological progression of depression by affecting neuroplasticity and neurogenesis (Duric et al., 2010; Yoshii and Constantine-Paton, 2010; Gormanns et al., 2011). MicroRNAs have an influence on depression by interfering with the stability of these signaling pathways (Fan et al., 2014).

**FIGURE 2:**
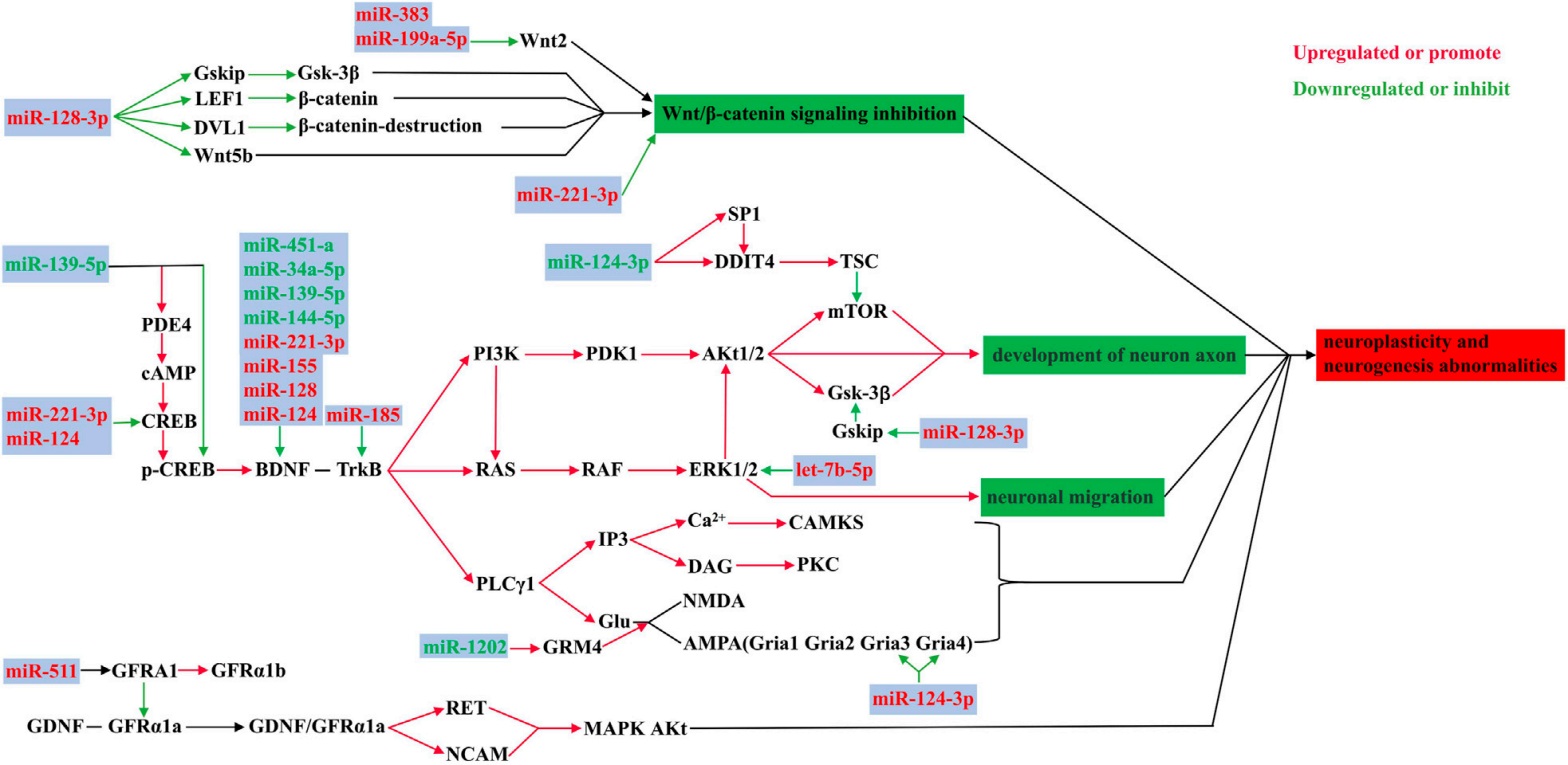
Role of microRNAs in neuroplasticity and neurogenesis abnormalities. MiR-124, miR-128, miR-139, miR-144 and others are involved in the regulation of neuroplasticity and neurogenesis through multiple pathways. These pathways mainly include Wnt/β-Catenin signaling pathway, mTOR signaling pathway, LTP signaling pathway, etc.

As shown in Figure 2. Wang et al. (2018b) found that miR-124-3p was significantly downregulated in Brodmann area 44 (BA44) of patients with MDD. Downregulation of miR-124-3p abolished its inhibition of DNA damage inducible transcript 4 protein (DDIT4) and SP1 expression, and inhibited the mTOR signaling pathway. Roy et al. (2020) demonstrated that miR-128-3p was upregulated in the amygdala of MDD patients, leading to a decreased expression of Wnt5b, LEF1 and DVL1, which are genes related to the Wnt signaling pathway. Disruption of canonical Wnt/Fz/GSK3 signaling leads to abnormal neurodevelopment that is associated with neuropsychiatric disorders (Voleti and Duman, 2012).

Moreover, the downregulation of Gria3 and Gria4 receptors induced by miR-124-3p had an influence on modulation of AMPA receptor, and correlated with an impaired synaptic plasticity in patients with depression (Roy et al., 2017a). In the basolateral amygdala of depressed patients, upregulated miR-511 downregulated the encoded GFRα1a specific isoform of the *GFRA1* gene of the receptor (Maheu et al., 2015). The subtypes, GFRα1a and GFRα1b elicited different downstream effects and had opposing effects in some aspects of neuroplasticity. The promotion of axonal growth by GFRα1a, was downregulated, while the inhibition of axonal growth by GFRα1b, was relatively upregulated, leading to the development of depression (Maheu et al., 2015). The upregulation of miR-185 in brain BA10 of MDD patients resulted in a decrease of TrkB-T1 expression. TrkB-T1, a BDNF receptor lacking the tyrosine kinase domain, was highly expressed in astrocytes and it regulated BDNF-evoked calcium transients (Maussion et al., 2012). Importantly, downregulation of TrkB-T1 in the frontal cortex might be associated with the neurobiology of suicide (Maussion et al., 2012).

In animal models, miR-139-5p regulates the cAMP/PKA/CREB/BDNF pathway to promote hippocampal neurogenesis by targeting PDE4D. Huang et al. (2021b) demonstrated that downregulation of miR-139-5p along with upregulation of its target gene PDE4D and downregulation of p-CREB and BDNF after inducing depression-like symptoms in CUMS mice. Such alterations show a bidirectional role for microRNAs in both protection and impairment of the neurogenesis pathways. In addition, Mingardi et al. (2021) found that miR-9-5p expression decreased in the hippocampus of rats subjected to chronic mild stress and primary hippocampal cultures. This change would cause overexpression of its downstream target protein REST, which would negatively affect neuronal dendritic morphology.

### 3.3 Role of microRNAs in MDD caused by changes of hypothalamic-pituitary-adrenal axis

Chronic stress has long been recognized to be a potential risk factor for depression, which is often associated with depression prevalence. The activity of the HPA axis is mediated by arginine vasopressin (AVP) and hypothalamic secretion of corticotropin releasing factor (CRF), which in turn activates the pituitary gland to secrete adrenocorticotropic hormone (ACTH), and finally stimulates the adrenal cortex to secrete glucocorticoids. Glucocorticoids then interact with receptors in multiple target tissues, where they directly exert negative feedback regulation on ACTH secreted by the pituitary as well as CRF secreted by the hypothalamus (Pariante and Lightman, 2008). Changes in glucocorticoid receptor (GR) expression, nuclear translocation, cofactor binding, and GR mediated gene transcription may play an important role in glucocorticoid resistance, which will lead to the development of HPA axis hyperactivity (Colla et al., 2007; Alt et al., 2010). Impaired GR function occurring in the periphery leads to the development of HPA axis hyperactivity. High glucocorticoid levels resulting from HPA axis hyperactivity may be involved in glucocorticoid-dependent hippocampal plasticity changes, causing hippocampal atrophy and reduced hippocampal neurogenesis, which in turn promotes the development of MDD (Kronenberg et al., 2009; Schmidt et al., 2009). As observed in depressed patients, HPA axis activity is the main biochemical change in addition to monoaminergic neurotransmitter disturbances (Budziszewska, 2002). MicroRNAs can influence the HPA axis activity by affecting glucocorticoid related receptors or other pathways (Uchida et al., 2008; Vreugdenhil et al., 2009).

Roy et al. (2017a) confirmed the effect of HPA axis hyperactivity on depression by examining the changes in miR-124-3p and its downstream target genes in PFC (BA46) and serum of mice with depression-like symptom after chronic CORT treatment. Furthermore, the detection of PFC (BA46) in post-mortem brains from depressed patients coincides with animal experiments (Roy et al., 2017a). In addition, upregulation of miR-124-3p in human and animal models was confirmed to be associated with downregulation of AMPA receptor family members Gria3 and Gria4, and glucocorticoid receptor NR3C1. MiR-124-3p mediated repression of NR3C1 may be central to the associated neuroendocrine response to stress (Roy et al., 2017a).

The central nervous system responses are of greater concern regarding hyperactive HPA axis responses. Al-Rawaf et al. (2021) demonstrated that the excessive cortisol activity induced by HPA axis hyperfunction was significantly correlated with decreased serotonin levels. A previous study has confirmed that the expression level of miR-124 was regulated by serotonin and demonstrated a significant negative correlation (Rajasethupathy et al., 2009). MiR-124 could control serotonin to induce synaptic function by repressing the transcription of cAMP response element binding protein (CREB), and conversely, CREB could further regulate miR-124 expression (Rajasethupathy et al., 2009). In addition, aberrant expression of miR-34a-5p and miR-451-a significantly reduced BDNF expression, and BDNF affected serotonin and cortisol expression by producing pro-neuroprotective signals (Numakawa et al., 2009; Numakawa et al., 2012; Wibrand et al., 2012).

### 3.4 MicroRNAs are involved in depression caused by abnormal inflammatory response

**FIGURE 3:**
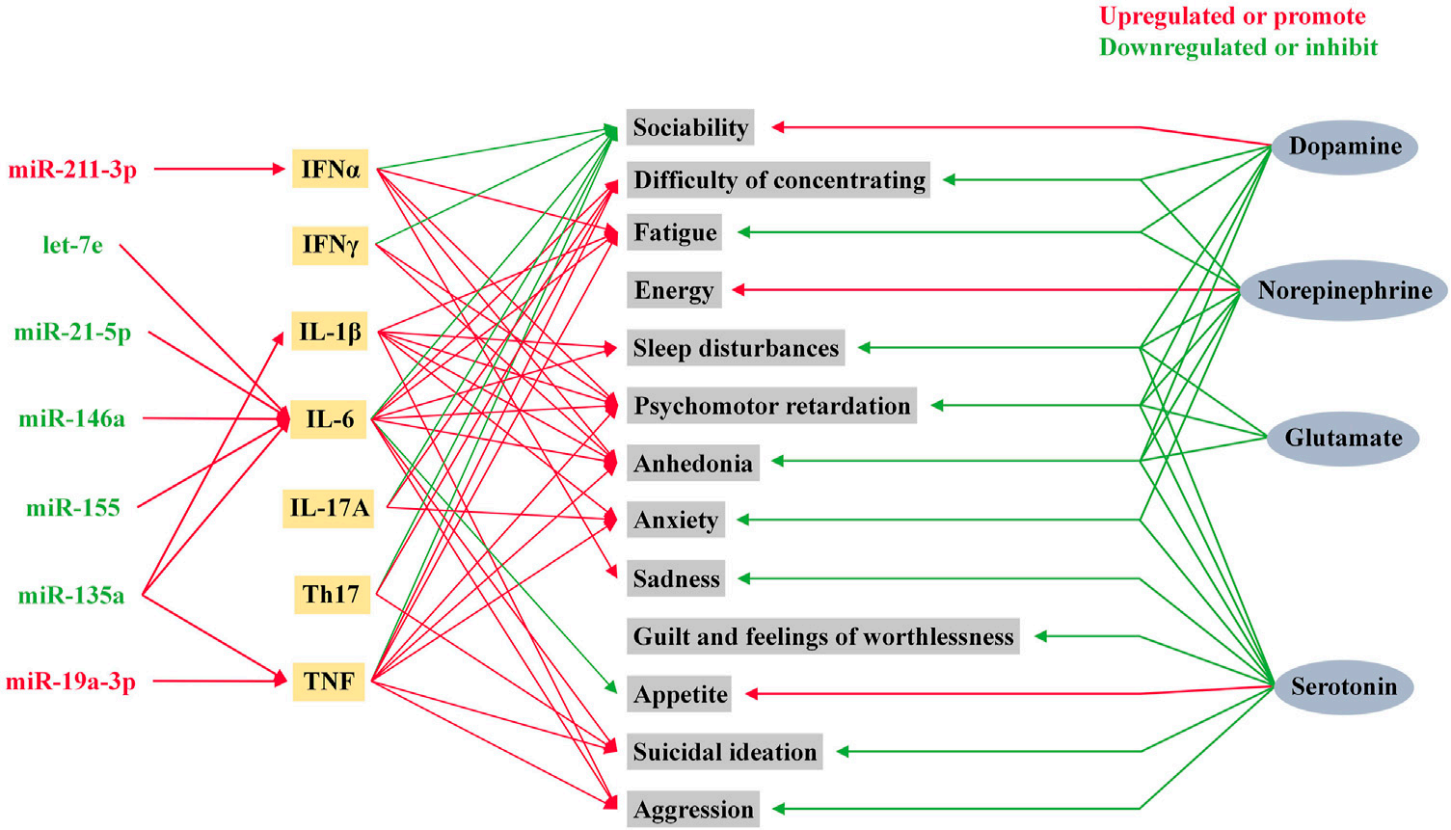
Role of microRNAs in inflammatory factors and neurotransmitters in depression. Psychiatric symptoms in humans are influenced by a variety of neurotransmitters and inflammatory factors. Among these inflammatory factors, IL-1β, IL-6, TNF, and IFNα are influenced by microRNA levels.

Depression and inflammation mutually contribute to the development of each other’s pathophysiology (Kiecolt-Glaser et al., 2015). Since the study of T and B lymphocytes in psychiatric patients by Herzog et al. (1979), the exploration of the relationship between the inflammatory response and depression has gradually unfolded (Herzog et al., 1979). Over the past four decades, accumulating evidence has shown that MDD is associated with systemic immune activation, including inflammatory markers, and changes in the number of immune cells (Gibney and Drexhage, 2013). Cytokines are one of the most important components of the immune system in depression. In response to peripheral infections, innate immune cells produce pro-inflammatory cytokines that act on the brain leading to development of neuropsychiatric disorders. When the peripheral immune system is continuously activated, immune signaling to the brain leads to exacerbation of the disease, and development of depressive symptoms in patients (Dantzer et al., 2008). The traditional routes of communication between the periphery and the central involve neural and humoral pathways, which mainly include: neural pathways (Harrison et al., 2009), signaling *via* cerebral endothelial cells (CECs) (Rivest et al., 2000; Kobayashi, 2010), signaling *via* circumventricular organs (CVOs) (Ransohoff et al., 2003) and peripheral immune-cell-to-brain signaling (Geissmann et al., 2003). TNFα, IL-1β and IL-6 are the main cytokines involved in the signaling of these pathways (Dantzer et al., 2008; Capuron and Miller, 2011). Recently, communication through the gut-microbiota-to-brain rout has gained increasing attention because of its role in regulating brain function (Jenkins et al., 2016; Sherwin et al., 2016). MicroRNAs participate in the pathophysiological process of inflammation in depression by promoting the production of inflammatory factors, as shown Figure 3. Changes in cytokine levels in patients with MDD have been identified to be associated with patient mood and volition (Beurel et al., 2020).

Wang et al. (2018a) pointed out that the upregulated expression of miR-19a-3p was detected in dlPFC and PBMC of MDD suicide completers. Gene analysis demonstrated that the elevated miR-19a-3p upregulated the expression of TNF-α by affecting the transcription of TAR-RNA binding protein (TRBP) and HuR (Wang et al., 2018a). The upregulation of TNF-α in dlPFC and PBMC was confirmed to be associated with suicidal ideation in MDD patients (Wang et al., 2018a). Sundquist et al. (2021) demonstrated that, in 178 patients with depression, anxiety, or stress and adjustment disorders, 36 inflammatory proteins with significantly different expression in peripheral blood of patients at baseline were seen, including 21 inflammatory proteins with increased levels and 15 with decreased levels, and all were associated with changes in miR-144-5p levels. In addition, the alteration in inflammatory proteins, which occurs after receiving treatment, was demonstrated to be associated with improvement in patients’ psychiatric symptoms (Sundquist et al., 2021). CircDYM, as an endogenous miR-9 sponge, is able to inhibit the activity of miR-9. Zhang et al. (2020b), by examining peripheral blood samples from MDD patients, hippocampus and plasma samples from MDD animal models, found that circDYM levels were significantly decreased. This would lead to enhanced miR-9 activity, which in turn would cause polarization of microglia. In a recent research, Xian et al. (2022) found miR-9-5p-enriched exosomes derived from PC12 cells in the serum of MDD patients. After BV2 microglia phagocytosed miR-9-5p-enriched exosomes, they were polarized to M1 subtype microglia *via* the SOCS2-STAT3 axis. Since then, M1 subtype microglia has produced a large amount of IL-1β, IL-6 and TNF-α. It leads to and intensifies the damage of neurons and causes the occurrence and development of MDD. Recent studies on depression triggered by microbial dysbiosis has shed new light on the role of abnormal inflammatory responses in the pathophysiology of depression (Borre et al., 2014; Dubois et al., 2019; Rea et al., 2020). This perspective explores the link between the gut microbiota and the regulation of the brain-gut axis, immune and endocrine system activity, and neurophysiological changes. Communication between the brain and the gut occurs bidirectionally *via* neural, endocrine, and immune pathways. Microbiota dysbiosis and an increased intestinal permeability with subsequent immune responses seem to be at the root of chronic mild inflammation associated with neuropsychiatric disorders (Petra et al., 2015; Rea et al., 2017; Farzi et al., 2018).

## 4 Summary and prospect

MicroRNAs are recognized as key epigenetic regulators of multiple functions in the brain and play a key role in MDD pathogenesis. As research continues to deepen, the roles of microRNAs in the pathophysiology of depression are gradually being elucidated. This review summarized recent research progress focusing on the role of microRNAs in the pathophysiology of depression, including dysregulation of monoamines, abnormalities in neuroplasticity and neurogenesis, hyperactivity of the HPA axis, and dysregulation of inflammatory responses. This suggests that an indispensable role for microRNAs occurs in these pathways. Several studies looking at changes in the levels of microRNAs and their downstream target genes before and after antidepressant treatment have confirmed a role for microRNAs in depression. Clearly, there are interactions between these different pathways and this exhibits the complexity in the pathogenesis of depression.

Based on the above four pathophysiological mechanisms of depression, it can be found that MDD, whether caused by dysregulation of monoamines or hyperactivity of the HPA, have parts that interact and influence each other. It is difficult to explain by a single pathophysiological mechanism, either from the clinical presentation of MDD patients or from changes in laboratory experiments. For example, high levels of cortisol in patients with Cushing syndrome resulted in alterations of neurotransmitter function, such as reduced serotonin synthesis. This can also be detected in MDD patients with HPA axis hyperactivity induced by long-term chronic stress (Stokes, 1995). In addition, high levels of cortisol inducing loss of hippocampal dendrites, and neuronal plasticity is recognized as one of the causes of depression (Gotlib et al., 2008). In addition, miR-124 can in turn control serotonin-induced synaptic facilitation by inhibiting the transcription of CREB (Rajasethupathy et al., 2009). Taken together, neuroinflammation could contribute to the pathogenesis of depression by interacting with the dysregulation of brain monoamines, dysregulation of the HPA axis, and alterations in hippocampal dentate gyrus neurons (Troubat et al., 2021).

It is important to note that current studies based on the role of microRNAs in depression have certain limitations, especially for the relationship between microRNAs and depression. Whether protective or injurious during the development of the disease, the levels of microRNAs in the brain tissue or peripheral tissues of patients do change when compared to normal individuals. Nevertheless, it is tough to confirm which of the varied microRNAs are responsible for the pathogenesis of MDD or that the major depressive disorder causes changes in certain microRNAs. If changes in specific microRNAs can be confirmed to contribute to the development of MDD, these microRNAs could be used as biomarkers for the diagnosis of the disease. In the same way, if it can be confirmed that MDD causes changes in the expression of microRNAs, and at the same time, alterations in these microRNAs can cause changes in the expression of downstream mRNAs and then have favorable or adverse effects on patients, this finding will be very important for the potential treatment of the disease and in stopping its development.

Since the discovery of the stable presence of free microRNAs in serum in 2018 (Chen et al., 2008), studies on the determination of microRNA levels in the serum of patients with depression have also gradually increased. However, it is undeniable that such studies have limitations as microRNAs in blood samples may not accurately reflect disease pathogenesis in the brain, because blood microRNAs are a mixture of brain-derived microRNAs and other microRNAs excreted from various tissues. The identification of microRNA within exosomes secreted by brain cells into the circulation may be able to compensate for the limitations that exist.

Finally, it is clear that microRNAs play an integral role in the pathophysiology of depression and may perhaps be able to provide a reference for the diagnostics and prognostics in depression by examining microRNA levels in relevant tissues. Moreover, promoting or inhibiting the expression of microRNAs might provide new clues for the development of antidepressant drugs.
